# Reviving Furosemide as a Metallo-&beta;-Lactamase Inhibitor against MDR *Acinetobacter baumannii*

**DOI:** 10.4014/jmb.2506.06023

**Published:** 2025-08-07

**Authors:** Hanan Al-Lawati, Fatma Al-Raisi, Mahmoud A. Elfaky, Mahmoud Saad Abdel-Halim, Hisham A. Abbas, Basem Mansour, Wael A. H. Hegazy, Noura M. Seleem

**Affiliations:** 1 Department of Pharmaceutics, Pharmacy Program, Oman College of Health Sciences, Muscat 113, Oman; 2 Department of Natural Products, Faculty of Pharmacy, King Abdulaziz University, Jeddah 21589, Saudi Arabia; 3 Centre for Artificial Intelligence in Precision Medicines, King Abdulaziz University, Jeddah 21589, Saudi Arabia; 4 Microbiology and Immunology Department, Faculty of Pharmacy, Zagazig University, Zagazig 44519, Egypt; 5 Pharmaceutical Chemistry Department, Faculty of Pharmacy, Delta University for Science and Technology, Gamasa 11152, Egypt; 6 Department of Pharmaceutical Chemistry, Faculty of Pharmacy, University of Kut, Kut, Wasit, 52001, Iraq; 7 Pharmacy Program, Department of Pharmaceutical Sciences, Oman College of Health Sciences, Muscat 113, Oman

**Keywords:** *Acinetobacter baumannii*, carbapenemases, metallo-β-lactamases, furosemide

## Abstract

Carbapenems are considered the last line of antibiotic defense against multidrug-resistant (MDR) *Acinetobacter baumannii*, a bacterium that can secrete carbapenemases such as metallo-β-lactamases (MBLs) to degrade carbapenems. It is thus critical to develop strategies to combat carbapenem resistance, and one of these strategies is to discover MBL inhibitors. In the current study, we evaluated the possible anti-β-lactamase of the approved safe drug furosemide. Furosemide is a loop diuretic with antihypertensive effects. A clinical MDR and carbapenem-resistant *A. baumannii* isolate was used. Furosemide at 1 mg/ml potentiated meropenem in the combined disk method and interfered with the hydrolytic activities of MBL. Furthermore, furosemide synergized meropenem and decreased its minimum inhibitory concentration (MIC). Furosemide could also reduce the expression of the MBL genes *bla*_NDM_ and *bla*_VIM_. In silico study showed the binding ability of furosemide with the active sites of New Delhi and VIM MBL enzymes, as well as its chelating interaction with zinc ions. Furosemide is a promising MBL inhibitor that can be used in combination with meropenem to treat infections caused by MBL-producing *A. baumannii*.

## Introduction

*Acinetobacter baumannii* is an opportunistic bacterium causing severe healthcare-associated infections [1, 2]. Along with an estimated one million cases of infection that occur each year, *A. baumannii* is also responsible for a high death rate, especially in critically ill patients [3-8]. This bacterium stands as one of the most perilous pathogens found in hospital settings, and is capable of inducing ventilator-associated pneumonia and bloodstream infections associated with central lines [3, 9, 10]. It exhibits elevated antibiotic resistance, which can manifest as multidrug resistance (MDR), extensive drug resistance (XDR), and pan-drug resistance (PDR) [6, 11, 12]. The rates of MDR in *A. baumannii* may reach up to four times higher than other major healthcare-associated bacteria [13], and may reach up to 45% of all *A. baumannii* isolates [13, 14]. Moreover, it is one of the ESKAPE pathogens that are considered as the most prominent nosocomial threats. ESKAPE pathogens include *Staphylococcus aureus*, *Enterococcus faecium*, *Pseudomonas aeruginosa*, *Klebsiella pneumoniae*, and *Enterobacter* spp. in addition to *A. baumannii* [15-18]. *A. baumannii* is also as a high-priority pathogen as classified by the World Health Organization (WHO) and the Centers for Disease Control and Prevention [19, 20].

Antimicrobial resistance (AMR) is a highly critical public health global crisis that may undermine the health progress achieved in the last century [21-24]. Horrifying estimates regarding antibiotic-resistant infections relate that they result in the deaths of 700,000 people yearly. If the current status of AMR continues, the number may increase to 10 million by 2050 [24-26]. Carbapenems are β-lactam antibiotics that are considered life-saving drugs used as a last resort for serious antibiotic-resistant infections caused by gram-negative bacteria [27, 28]. The primary resistance mechanism to carbapenems is mediated by carbapenem-inactivating enzymes known as carbapenemases [27, 29, 30]. There are two categories of enzymes responsible for such inactivation: the first are called serine β-lactamases, which operate through a serine residue; those in the second category, termed metallo-β-lactamases (MBLs), are mediated by one or two zinc ions [27]. Only certain serine β-lactamases, such as *K. pneumoniae* carbapenemases (KPCs) and oxacillinase-48 (OXA-48-like) carbapenemases, possess the capability to hydrolyze carbapenems [31, 32]. In contrast, all MBLs can hydrolyze carbapenems [33, 34]. The concern surrounding MBL is heightened by the absence of clinically effective MBL inhibitors [35, 36].

MBLs are classified into three subclasses (B1, B2, and B3) based on their metal content and distinctive active site features [34, 37]. The majority of MBLs belong to the B1 subclass, such as the imipenemase (IMP), New Delhi-MBL (NDM), and Verona imipenemase (VIM) families, which include the most frequent metallo-β-lactamases present in clinical isolates [38]. To counteract β-lactamase-mediated resistance, two approaches are employed. One approach depends on the synthesis of novel β-lactamase-resistant β-lactams, such as cefiderocol, while the second is based on the production of β-lactamase inhibitors for use in combination with antibiotics [39, 40]. Unfortunately, clinically approved inhibitors for MBL are currently unavailable [41].

Furosemide is one of the FDA-approved loop diuretics to treat volume overload and edema associated with congestive heart failure in addition to hepatic or renal failure [42]. Bearing in mind its chemical structure, furosamide exhibits different pK values and can form complex compounds with various metal ions [43, 44]. On this basis, we were inspired to investigate the possible inhibitory effect of furosemide against MBL production in *A. baumannii* by in vitro and in silico studies.

## Materials and Methods

### Materials

All the antibiotic discs and media used were purchased from Oxoid (UK). Furosemide and EDTA were also ordered from Sigma-Aldrich (USA). Meropenem powder was obtained from AstraZeneca (UK).

### Bacterial Isolate

A multi-drug resistant and carbapenem-resistant *A. baumannii* isolate was sourced from the stock culture of the Department of Microbiology and Immunology, Faculty of Pharmacy, Zagazig University. It was separated from a bronchial aspirate from an ICU patient and was previously characterized as an NDM-1 and VIM producer. The isolate is resistant to piperacillin, ceftriaxone, imipenem, ceftazidime, cefepime, meropenem, ciprofloxacin, amikacin, and sulfamethoxazole-trimethoprim.

### Evaluation of Minimum Inhibitory Concentration (MIC) of Meropenem and Furosemide against Gram-Negative Strains

To determine the MICs of meropenem and furosemide, the broth microdilution method was used [17, 45].

### Effect of Furosemide on Bacterial Growth

To ensure that the potential effect of furosemide on MLB was not due to its growth- inhibiting activity, the effect of the sub-inhibitory concentration of furosemide on the growth of the tested *A. baumannii* strain was detected and compared to the bacterial growth in the absence of furosemide [46, 47].

### Detection of MBL Production by the Combined EDTA-Disk Synergy Test

To detect whether the tested strain produces MBLs, the imipenem inhibition zone augmentation in the presence of EDTA (pH 8.0) was determined by the combined EDTA-disk synergy test [48, 49]. An overnight liquid culture with a turbidity matching that of a 0.5 McFarland standard was prepared, and aliquots of 100 μl were spread on the surfaces of a Mueller-Hinton agar plate. Two meropenem disks were put on the center of each of the test and control plates, and the inhibition zone diameters were measured after incubation at 37°C for 20 h.

### Quantitative Inhibition Assay of Carbapenemase

The crude periplasmic extract of *A. baumannii* isolate was prepared following Bernabue *et al*. [50]. Mueller-Hinton broth (10 ml) was inoculated with a loopful of *A. baumannii* culture and then incubated for 20 h at 37°C with shaking. The pellets were collected by centrifugation and then resuspended in 0.5 ml of phosphate buffer provided with 50 μM ZnSO4. The pellets were sonicated (Ultrasonic System UP100H, Hielscher–Ultrasound Technology, Germany) at 40W for 45 s. After centrifugation, the supernatants were obtained to determine the hydrolysis of meropenem in the presence and absence of 1 mg/ml furosemide at a wavelength of 297 nm using a UV-Vis spectrophotometer (Synergy HT, BioTek, USA) following the modified protocol of Denny *et al*. [51]. To aliquots of 100 μl of the supernatants, 1 mg/ml furosemide was added for 30 min at 37°C, followed by the addition of meropenem (500 μg/ml), for 60 min at 37°C. The absorbances at 297nm (A_297_) of furosemide-treated and control solutions were measured. The percentage of inhibition of meropenem hydrolysis was calculated using the following formula:

% of inhibition = [(A_297_ test—A_297_ vehicle control)/ A_297_ test] × 100.

The half minimal inhibitory concentration (IC_50_) represents the minimum concentration of furosemide needed to inhibit the activity of the extracted carbapenemases by 50%. To determine this, four concentrations of furosemide (0.25, 0.5, 1, and 2mg/ml) were employed to assess the percentage of carbapenemase inhibition, from which the IC_50_ value was calculated.

### Synergy Testing of Meropenem by Sub-MIC of Furosemide

The MIC of meropenem was evaluated in the presence of sub-MIC (1 mg/ml) concentrations of furosemide, employing the broth microdilution method as previously outlined [17, 52, 53].

### Downregulation of Metallo β-Lactamase Genes by Quantitative RT-PCR

The relative expression levels of MLB genes *bla*_NDM_ and *bla*_VIM_ in *A. baumannii* treated with or without furosemide were detected by quantitative qRT-PCR. The relative expressions of the tested genes were normalized to the housekeeping gene 16s rRNA [54-56]. The StepOne Real-Time PCR system (Applied Biosystems, USA) was employed following the SensiFAST SYBR Hi-ROX One-Step Kit protocol (Bioline, UK). Gene expression levels were assessed using the comparative threshold cycle ^(ΔΔCt)^ method [57-59]. The primers used are listed in Table 1.

### In Silico Study

The crystal structures of the NDM-1 and VIM-2 MBLs (PDB ID: 3SPU and PDB ID: 5YD7, respectively) were retrieved from the Protein Data Bank (https://www.rcsb.org/) in PDB format. Each of the meropenem and furosemide structures was drawn with MarvinSketch of the Marvin Suite (http://www.chemaxon.com) in order to generate a three-dimensional conformer for each, with the least energy, and then saved in the Mol2 format [60, 61]. The crystal 3D structures of the MLB enzymes were protonated with their standard geometry after removing all water molecules, followed by minimization of energy. The tested compounds were docked into the rigid binding pocket of the proteins using flexible ligand mode. From ligand conformations, the placement phase generates poses. The GBVI/WSA ΔG was used to estimate the free energy of binding of the ligand from a given pose as a force field-based scoring function [62].

### Statistical Analysis

Student’s *t*-test was used to detect the significance of the effects of furosemide against growth, meropenem synergy, and carbapenemase inhibition. The one-way ANOVA and Dunnett’s post-hoc test were employed to determine the significance on downregulation of MBL genes by quantitative real-time PCR, and the statistical significance was *p* < 0.05.

## Results

### MICs of Meropenem and Furosemide

The MIC determination results for meropenem and the test drug furosemide against *A. baumannii* showed growth inhibition of 1,024 μg/ml and > 5 mg/ml, respectively. For all subsequent experiments, the sub-inhibitory concentration of 1 mg/ml of furosemide was used.

### Furosemide Has No Effect on the Growth of *A. baumannii*

**Fig. 1:**
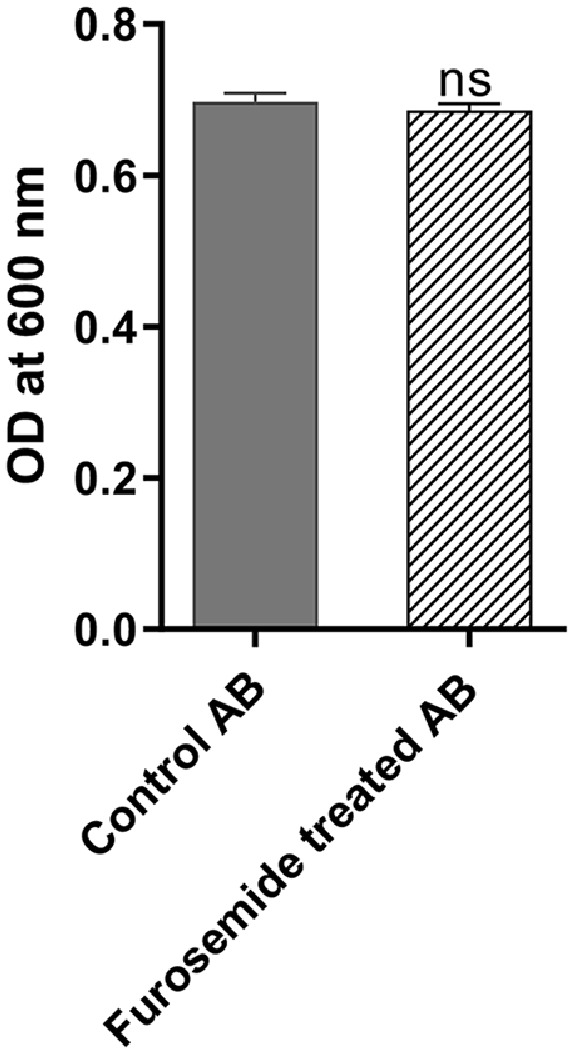
Effect of furosemide on the growth of A.baumannii. No significant difference between the growth in control and treated cultures was found, ns indicates no significant difference.

To have an effect on the metallo-β-lactamases of *A. baumannii*, the anti-enzyme activity of a test drug must be away from its effect on growth. To ensure this, the growth-inhibiting activity of furosemide was investigated. The optical densities (ODs) of the bacterial suspensions treated and untreated with sub-inhibitory concentrations of furosemide were compared. As no significant differences in the ODs were found between the control and the treated suspensions, we assumed that furosemide does not affect the growth (Fig. 1).

### Furosemide Potentiated Meropenem in the Combined Disk Test

**Fig. 2:**
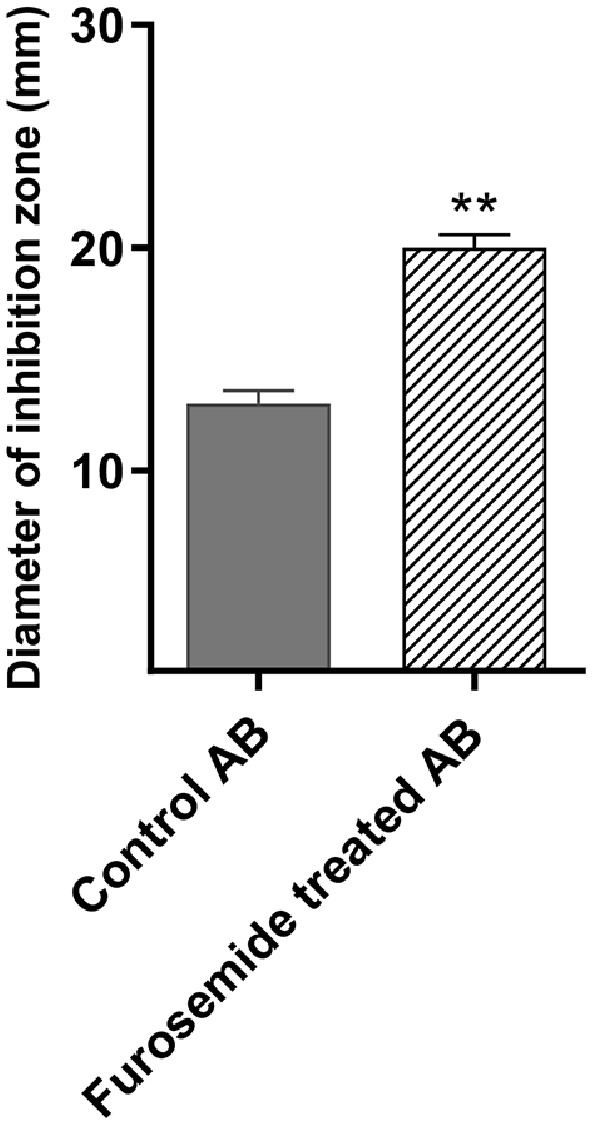
Furosemide synergized meropenem antibacterial activity in the combined disk test. Meropenem inhibition zone was significantly increased in furosemide containing plates.

To test whether there was any synergy between meropenem and furosemide, the combined disk test was used. There was a significant increase in the inhibition zone of meropenem in the furosemide-containing plates as compared to the control plates (Fig. 2).

### Furosemide Interfered with the Hydrolytic Activity of Carbapenemases in the Crude Periplasmic Extract of *A. baumannii*

**Fig. 3:**
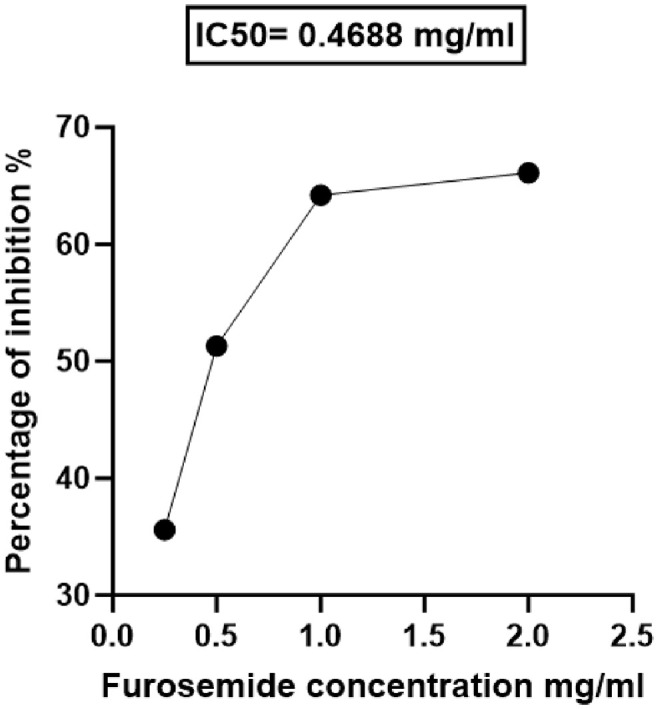
Inhibition of carbapenemase by furosemide. Sub-inhibitory concentration of furosemide inhibited the activity of carbapenemases in a dose-dependent manner.

To investigate the possible inhibition of carbapenemase-mediated meropenem hydrolysis, the absorbances of meropenem in the crude periplasmic extract of the *A. baumannii* isolate treated and untreated with furosemide were measured. Furosemide showed a significant diminishing effect on the metallo-β-lactamase activity when co-incubated in a culture supernatant in a concentration-dependent matter. The furosemide IC_50_ for inhibition of carbapenemase hydrolytic activity was 0.4688 mg/ml (Fig. 3).

### Furosemide Augmented the Antibacterial Activity of Meropenem against *A. baumannii*

To assess the possible synergy between meropenem and furosemide, the MIC of meropenem was either in combination with furosemide or not. Furosemide increased the susceptibility of *A. baumannii* to meropenem by 16-fold as shown in Table 2.

### Furosemide Decreased the Relative Expression of MBL Genes *bla*_VIM_ and *bla*_NDM_ in *A. baumannii*

**Fig. 4:**
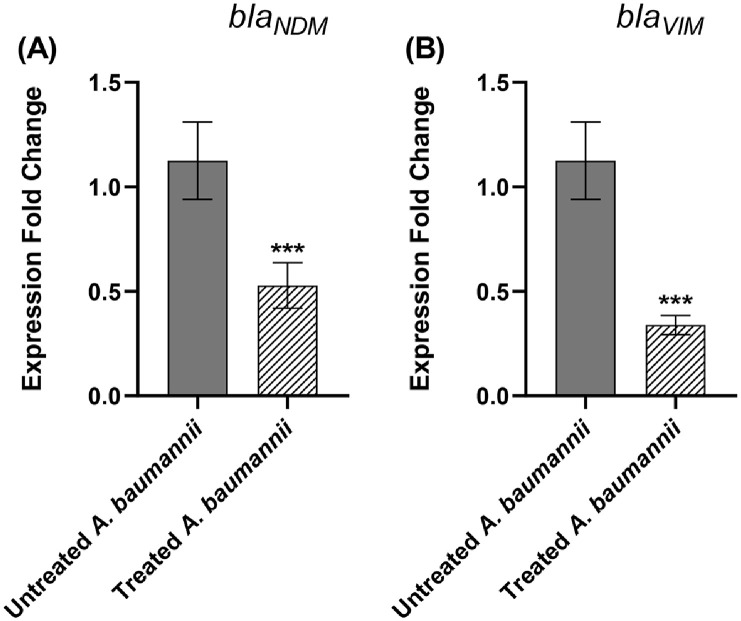
Reduced relative expression of the genes *bla*_VIM_ and *bla*_NDM_ genes by furosemide. Furosemide downregulated the expression of both genes.

The effect of furosemide on the gene expression of metallo-β-lactamase genes *bla*_VIM_ and *bla*_NDM_ in the tested *A. baumannii* strain was further investigated at the molecular level by quantitative real time PCR. Interestingly, furosemide downregulated the expression of *bla*_NDM_ and *bla*_VIM_ by 50% and 60%, respectively (Fig. 4).

### Furosemide Could Bind to the Active Sites of New Delhi and VIM Metallo-β-Lactamases in the in Silico Study

The in silico or molecular modeling simulation is a useful tool in mimicking the behavior of molecules and molecular systems since the nature of the interactions between ligands and macromolecules or receptors is of special interest [63]. Furosemide (4-chloro-2-[(2-furylmethyl) amino]-5-sulfamoylbenzoic acid) is characterized by its plentiful H-bond donor and/or acceptor sites, which play a major role in binding to the receptor active site [64].

**Fig. 5:**
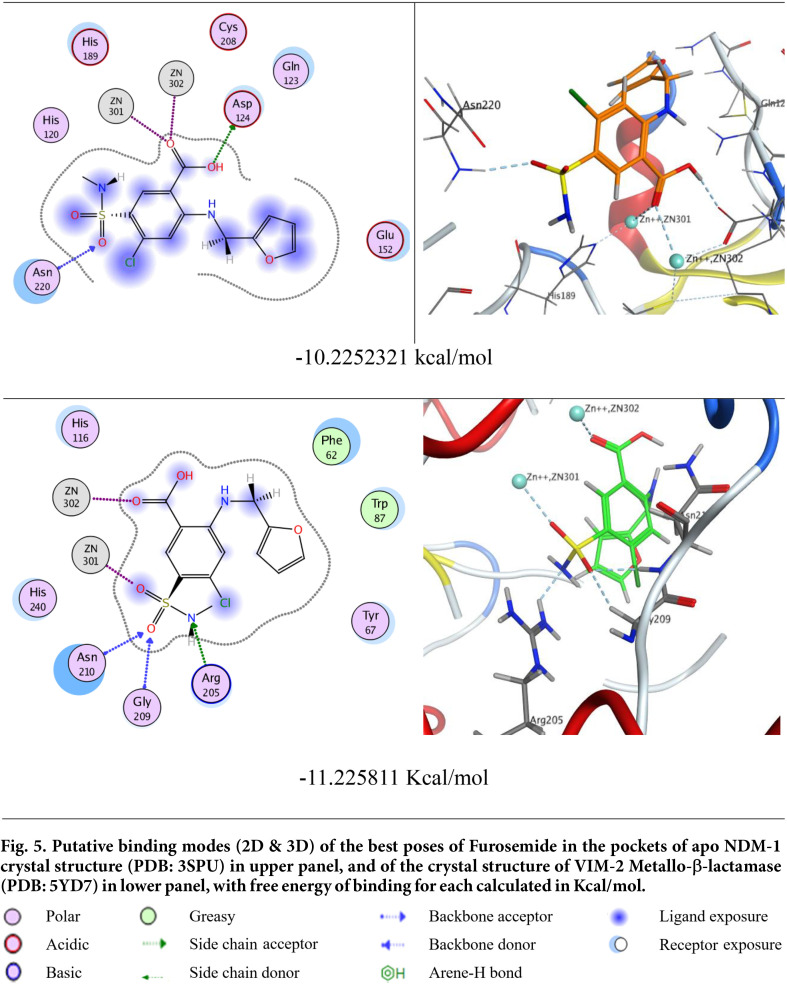
Fig. 5

As illustrated in Fig. 5, the docking results of the furosemide ligand against the crystal structure of the New Delhi metallo-β-lactamase-1 revealed that the carboxylic hydroxyl via its oxygen atom could form a H-bond with the H-bond acceptor side chain of the conserved amino acid Asp124. Furthermore, the *sp2* hybridized oxygen atom in the carboxylic group has two lone pairs that can act as Lewis bases, so it can coordinate with the center of two positively charged divalent ions, Zn^2+^301 and Zn^2+^ 302, acting as Lewis acids [65]. On the other hand, in the sulfamoyl group one of the two *sp2* hybridized oxygen atoms acted as a H-bond acceptor to form a H-bond with the H-bond donor backbone of the conserved amino acid Asn220, thus stabilizing the ligand/ receptor complex to achieve binding free energy of -10.2252321 kcal/mol.

Concerning the docking against the crystal structure of VIM-2 metallo-β-lactamase, we found that the sulfamoyl group played a significant role in fixation of the ligand in the core of the active site of the receptor; its primary amine accepted a H-bond from the H-bond donor side chain of the conserved amino acid Arg205, and while one of its *sp2* hybridized oxygen atoms formed a bifurcated H-bond with the H-bond donor backbones of Gly209 and Asn210, the other was coordinated by Zn^2+^301. Moreover, the sp2 hybridized oxygen of the carboxylic group exhibited a coordinate covalent bond, or simply coordinate bond, with the center of Zn^2+^ 302. In addition, the hydrophobic-hydrophilic interaction observed through the blue and cyan shadows around ligand moieties and amino acids of the receptor improved the overall recognition of the ligand inside the hot spot of the active site leading to a free binding energy of -11.225811 Kcal/mol.

It is noteworthy that the two carboxylic and sulfamoyl functionalities of furosemide were not only of convenient size but were also very essential for ligand-receptor interactions due to having plenty of H-bond donor and/or acceptor sites. Being Lewis bases, the sp2 hybridized oxygen atoms in the carboxylic or sulfamoyl moieties were very appropriate chelating centers for Zn^2+^301 and Zn^2+^302. Given that zinc(II) is not just a constituent of metalloenzymes, but also has a catalytic function, upon zinc(II) chelation, these enzymes will therefore be inhibited [66].

## Discussion

*A. baumannii* is an important healthcare-associated pathogen due to its ability to persist in hospitals armed by multidrug-resistance to antibiotics and the possibility of colonization of susceptible patients who are under broad-spectrum antibiotic therapy [67]. This bacterium is responsible for a plethora of infections, such as those affecting the urinary tract, skin and soft tissues, in addition to bacteremia and pneumonia, particularly in immunosuppressed patients [68-70]. β-Lactam antibiotics are life-saving antibiotics that are still among the most common antibiotics used clinically. As a result, the rapidly increasing emergence of resistance against β-lactam antibiotics is a great and worrisome public health problem globally [12, 71, 72]. Being the best drug for treating nosocomial infection caused by MDR *A. baumannii*, it is clear how big the problem of *A. baumannii*’s resistance to carbapenems is, especially considering that it is worsening worldwide [73].

The primary mechanism of resistance to carbapenems is via carbapenemases, which include metallo-*β*-lactamases (MBL). In *A. baumannii*, different types of MBLs, such as NDM-type, and VIM-like carbapenemases are present [74, 75]. Due to the importance of carbapenems in treating resistant *A. baumannii* infections, it is of extreme importance to re-sensitize carbapenems by combining them with carbapenemase inhibitors. There are approved carbapenemase inhibitors such as vaborbactam for pneumonia, and combined with meropenem for complicated urinary tract infections, but vaborbactam lacks the efficacy against Class B or D carbapenemases [76, 77]. Moreover, relebactam is used in combination with imipenem, but relebactam lacks the activity against Class D OXA-48 β-lactamases [78].

In this study, a clinical *A. baumannii* isolate that was multidrug resistant and carbapenem resistant was used. Furosemide was used at a sub-inhibitory concentration that was found to have no inhibitory activity against bacterial growth, so any inhibitory effect on MBLs is away from the effect on cell growth. Furosemide potentiated meropenem in the combined disk method and increased the diameter of the inhibition zone produced by meropenem. Moreover, the susceptibility to meropenem was enhanced by furosemide as demonstrated by a 16-fold decrease in MIC. On quantitative assay of the inhibition of carbapenemase-mediated hydrolysis of meropenem, furosemide could protect meropenem against hydrolysis. At the molecular level, furosemide significantly reduced the expression of two MBL genes, *bla*_NDM_ and *bla*_VIM_ in quantitative real-time PCR. This provides strong confirmation of the inhibitory activity of furosemide against MBLs.

We hypothesize that furosemide inhibits MBLs by chelation of zinc ions that are essential for the activity of the enzymes. There are many previous reports about compounds that could inhibit different MBLs. These compounds have some functional moieties that enable them to chelate metals in MBLs, such as thiols, thiones, triazoles, sulfonic acids, carboxylic acids and hydroxamates [79]. Examples of these compounds are captopril and dimercaprol [80]. Furosemide or 4-chloro-2-(furan-2-ylmethylamino)-5-sulfamoylbenzoic acid has the carboxylic acid moiety and the sulphamoyl moity that may account for its ability to chelate zinc ions [81]. We conducted a docking study to investigate this hypothesis. Furosemide could bind with New Delhi MBL-1 by means of hydrogen bonding formed between the oxygen atom of the carboxylic hydroxyl and the H-bond acceptor side chain of Asp124, as well as the coordination between the *sp2* hybridized oxygen atom in the carboxylic group with its two lone pairs and the center of Zn^2+^301 and Zn^2+^ 302. This is in addition to the hydrogen bond between the sulfamoyl group *sp2* hybridized oxygen atoms and the H-bond donor backbone of Asn220. Furthermore, furosemide could bind to VIM-2 MBL by hydrogen bonding between the sulfamoyl group and the amino acid Arg205, and coordination between its *sp2* hybridized oxygen atoms and Zn^2+^301. Moreover, the carboxylic group *sp2* hybridized oxygen can form a coordinate covalent with Zn^2+^ 302.

In a nutshell, we investigated the β-lactamase inhibition potential of furosemide, a widely prescribed diuretic. Repurposing furosemide as a safe, approved agent (at low concentration of 1 mg/ml) could offer significant advantages for treating infections caused by carbapenemase-producing bacteria like *A. baumannii*, especially given the limitations of current inhibitors. For example, vaborbactam lacks efficacy against Class B and D carbapenemases [48, 49], and relebactam is inactive against class D OXA-48 β-lactamases [50]. Furthermore, furosemide’s potential extends beyond systemic use; since *A. baumannii* frequently causes topical infections (*e.g.*, burns, wounds) [82], and furosemide has been demonstrated to be safe for topical application, such as in an intranasal spray [83], it holds promise for topical treatment of wound/burn infections involving MBL-producing *A. baumannii*. Finally, confirming furosemide's β-lactamase inhibition opens avenues for designing novel pharmacophores as next-generation β-lactamase inhibitors. This study represents a preliminary investigation. In future work, we plan to expand our research scope to include broader clinical isolate testing and assessment of furosemide’s efficacy in combination with other β-lactam antibiotics, accompanied by comprehensive pharmaceutical and pharmacological analyses.

## Conclusion

Furosemide is a promising MBL inhibitor in *A. baumannii*. This anti-enzyme activity enables the use of furosemide as a combination therapy with carbapenems to carbapenem-resistant infections caused by *A. baumannii*. The furosemide activity could be attributed to its ability to downregulate the carbapenemase-encoding genes and possible interference with the enzyme itself. The current study paves the way to screen and construct the drugs and the compounds with similar moieties to serve as MBL inhibitors. However, the investigation should be expanded to include further pharmaceutical and pharmacological studies to assure clinical effectiveness and safety.

## Figures and Tables

**Table 1:** Primers used.

Gene Primers	Sequences (5' → 3')
*VIM*	F = 5' GAT GGT GTT TGG TCG CAT A 3'
	R = 5' CGA ATG CGC AGC ACC AG 3'
*NDM*	F = 5' GGT TTG GCG ATC TGG TTT TC 3'
	R = 5' CGG AAT GGC TCA TCA CGA TC 3'
*16s rRNA*	F = 5' TCA GCT CGT GTC GTG AGA TG 3'
	R = 5' CGT AAG GGC CAT GAT G 3

**Table 2:** Synergistic effect of meropenem and furosemide against *A. baumannii*.

MIC (μg/ml)	
Meropenem	Meropenem + furosemide (1 mg/ml)
1024	64
